# Concerns of orthodontic patients during the COVID-19 quarantine period

**DOI:** 10.1590/2177-6709.27.1.e2220229.oar

**Published:** 2022-04-11

**Authors:** Rodrigo NAVEDA, María Pía SEMINARIO, Guilherme JANSON, Daniela GARIB

**Affiliations:** 1 Universidade de São Paulo, Faculdade de Odontologia de Bauru, Departamento de Ortodontia (Bauru/SP, Brazil).; 2 Universidade de São Paulo, Hospital de Reabilitação de Anomalias Craniofaciais (Bauru/SP, Brazil).

**Keywords:** Quality of life, Orthodontics, General health

## Abstract

**Introduction:**

Quarantine protocols for coronavirus disease 2019 (COVID-19) pandemic has modified orthodontic appointments.

**Objective:**

to evaluate self-reported experience and needs of orthodontic patients during the quarantine period without in-person appointments.

**Methods:**

Thirty patients, aged 8 to 21 years, under active orthodontic treatment were randomly selected. A phone call questionnaire including questions on physical and emotional impacts of the quarantine of COVID-19 was applied during the second month of Brazilian quarantine.

**Results:**

Sixteen percent of the patients reported pain related to the orthodontic appliance. Appliance breakage was observed in 23.33%. Twenty percent felt the need of an emergency orthodontic appointment and 3.33% visited a private practice. Moreover, 23% reported that even in an emergency need, they would not search for an appointment, preferring to communicate with the orthodontist by WhatsApp. Oral hygiene self-perception status showed improvement in 36.67% and worsening in 6.67% of the patients, while 56.66% reported maintenance of the same hygiene status. Sixty percent were concerned about the orthodontic treatment. General concerns were primary related to health and a possible extension of the quarantine time.

**Conclusions:**

During COVID-19 quarantine, orthodontic patients demonstrated a low frequency of orthodontic appliance interference in daily life, and most of them reported a maintenance of oral hygiene habits. The frequency of appliance related pain and breakage was 16.67% and 23.33%, respectively. Patients’ main concern during the quarantine period was the fear of getting sick and the uncertainness about the extension of the quarantine period.

## RESUMO

**Introdução:**

As medidas de quarentena para a pandemia da doença coronavírus 2019 (COVID-19) modificaram as consultas ortodônticas.

**Objetivo:**

O objetivo desse estudo foi avaliar as experiências e necessidades autorreferidas dos pacientes ortodônticos durante o período de quarentena sem consulta ortodôntica presencial.

**Métodos:**

Trinta pacientes, com idades entre 8 e 21 anos, em tratamento ortodôntico ativo, foram selecionados aleatoriamente. Um questionário com perguntas relacionadas aos impactos físicos e emocionais durante a quarentena de COVID-19 foi aplicado durante o segundo mês de quarentena brasileira, por meio de ligações telefônicas.

**Resultados:**

Dezesseis por cento dos pacientes referiram dor relacionada ao aparelho ortodôntico, e a quebra do aparelho foi observada em 23,33%. Vinte por cento sentiram necessidade de consulta ortodôntica de emergência, 3,33% procuraram atenção particular e 23% disseram que, mesmo diante de uma emergência ortodôntica, não procurariam atenção particular e prefeririam se comunicar com o ortodontista pelo WhatsApp. A autopercepção do estado de higiene bucal apresentou melhora em 36,67% e piora em 6,67% dos pacientes, enquanto 56,66% relataram manutenção do mesmo estado de higiene. Sessenta por cento estavam preocupados com o tratamento ortodôntico. As preocupações gerais estavam relacionadas com a saúde e uma possível extensão do tempo de quarentena.

**Conclusões:**

Durante a quarentena de COVID-19, os pacientes ortodônticos demonstraram baixa frequência de interferência do aparelho ortodôntico no dia a dia, e a maioria relatou manutenção dos hábitos de higiene bucal. A frequência de dor relacionada ao aparelho e de quebra do aparelho foi de 16,67% e 23,33%, respectivamente. A principal preocupação dos pacientes durante o período de quarentena era ficar doente, além do medo de uma extensão do período de quarentena.

## INTRODUCTION

The first pandemic of the 21^st^ century started in Wuhan, Hubei Province, China, causing the coronavirus disease 2019 (COVID-19).^1^ This situation has changed the lifestyle around the globe. Mental health and quality of life have been affected by the fear of the new coronavirus, and by the quarantine protocols that were taken to decrease the contagious rate.^2^

COVID-19 has caused a worldwide socioeconomic impact, decreasing the monthly income of people around the world.^3^ Transmission mechanisms have affected several professions,^4^ ^,^ ^5^ and it is assumed that dentistry presents a high infection risk, mainly due to direct contact with patient’s fluids during appointments and to possible cross-infection.^4^ ^,^ ^5^

Quarantine protocols for contention of COVID-19 pandemic has paused orthodontic monthly appointments in two or four months.^6^ Periods between orthodontic consultations vary depending on the type of treatment. In general, a 28-day interval showed great results, especially for fixed appliances.^7^ Longer interval periods might be detrimental for dental and periodontal health, and emergencies vary when using removable or fixed appliances.^7^ Patients with skeletal anchorage might have increased risk of emergencies.^8^

Previous study reported that patients showed good response in managing orthodontic appliances-related physical, practical and emotional impacts.^9^ However, the quarantine period increased the risks of psychological problems,^2^ ^,^ ^10^ affecting how patients respond to orthodontic treatment. Therefore, the objective of this study was to evaluate the self-reported perceptions and needs of orthodontic patients during the quarantine period without in-person appointments.

## MATERIAL AND METHODS

This cross-sectional, qualitative study was approved by the institutional Research Ethics Committee of *Faculdade de Odontologia de Bauru, Universidade de São Paulo* (process nº 32021020.7.0000.5417). Subjects were selected from the clinic list of patients of the Orthodontic Department of Orthodontics of the aforementioned institution. Fifty-eight patients (age between 8 and 21 years) under active orthodontic treatment were contacted via WhatsApp message by a faculty member. Patients with chronic medical conditions and craniofacial anomalies were excluded. An invitation letter was sent to patients explaining the objective of the study, and the ones with a positive response were included. Thirty patients/parents accepted to participate and were enrolled in this survey. The sample was composed by 14 males and 16 females with a mean age of 14.17 years (SD = 3.03). From the complete sample, 29 patients had conventional fixed appliances and 1 had only a fixed maxillary expander. 

**Figure 1:**
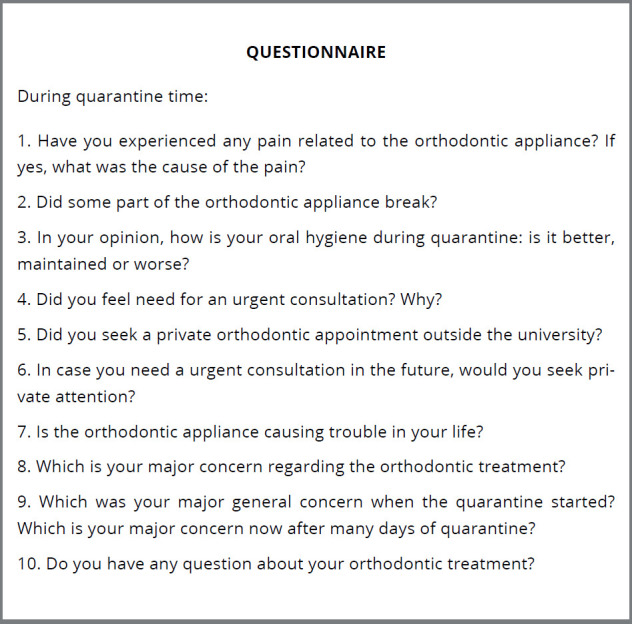
Questionnaire.

Patients were contacted via WhatsApp by their orthodontists monthly during the extended quarantine and were encouraged to contact them back in case of queries or emergency need. Patients were contacted after 60 to 80 days from the last orthodontic appointment by the study interviewer. It is important to highlight that the interviewer was not their own orthodontist. A phone call questionnaire approaching physical and emotional impacts of the quarantine was applied during the second month of Brazilian quarantine. The questionnaire was composed by 10 objective/subjective questions covering pain, appliance breakage, oral hygiene status self-perception, emergency appointments need and fear/concerns (Fig 1). Support was given by the interviewer when patients did not understand the question. Regarding subjective questions, no answer options were offered and the responses were freely provided by patients or guardians. Answers to subjective questions were then grouped based on their similarities. 

 Patients under 18 years of age were interviewed accompanied by their parents. All interviews were recorded and transcribed into a Microsoft Word document for analysis.

### STATISTICAL ANALYSIS

Descriptive analysis were performed using Statistica software (Statistica for Windows, version 11.0, Statsoft, Tulsa, Okla).

## RESULTS

**Figure 2:**
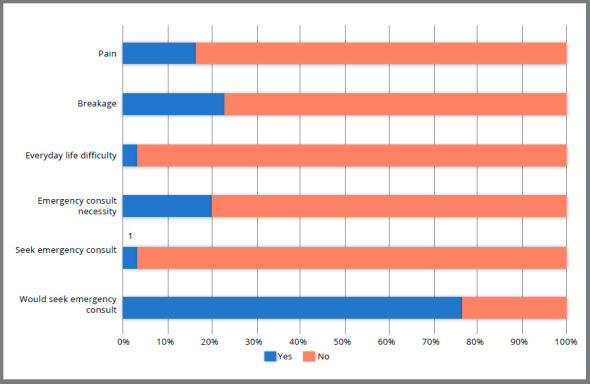
Orthodontic appliance pain, breakage, other daily life difficulties and emergency appointment frequencies.

From the complete sample, 5 patients (16.67%) reported pain related to the orthodontic appliances during quarantine (Fig 2). Pain was related with orthodontic bands (40%), intermaxillary elastics use (20%) and orthodontic archwire displacement (40%). Fixed appliances breakage was reported by 7 patients (23.33%) and was associated with orthodontic bands (14.29%), bracket debonding (28.57%), orthodontic archwire fracture (14.29%) and broken elastics (42.85%). Only 1 patient (3.33%) reported that daily life was difficult due the orthodontic appliances during quarantine. 

**Figure 3:**
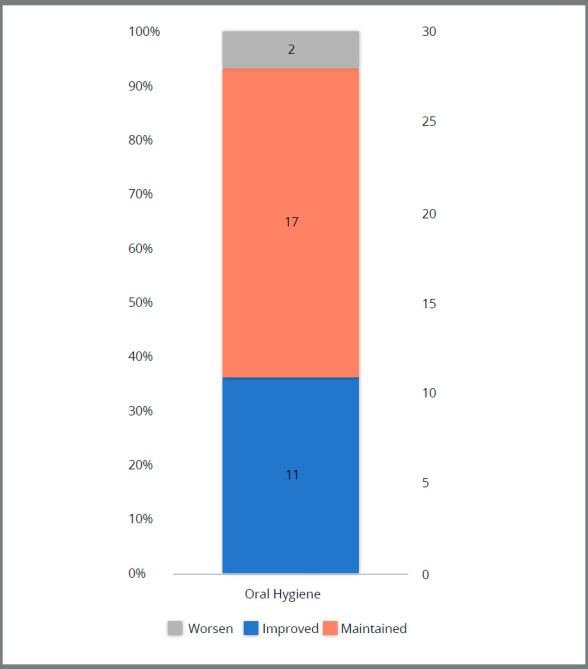
Oral hygiene self-perception status.

Twenty percent of the patients felt the need of an emergency appointment, even though only one patient (3.33%) visited a private practice outside the university (Fig 2). Twenty-three percent of the subjects reported that they would not look for an appointment even in case of emergency, preferring to communicate with the orthodontist via WhatsApp. Oral hygiene self-perception status showed improvement in 36.67% and worsening in 6.67% of the patients, while 56.66% reported maintenance of the hygiene status as before the quarantine (Fig 3).

Eighteen subjects (60%) were concerned about the orthodontic treatment (Table 1). Concerns were related to pain (16.67%), appliance breakage (16.67%), appointment need (22.22%), oral hygiene status (16.67%) and increased treatment time (27.77%). General concerns at the beginning of the quarantine and at the moment of the interview were also explored. The main concern at the quarantine start was the own fear or someone in the family getting sick with Coronavirus, observed in 36.6% and 23.3% of the participants, respectively. At the moment of the interview, 13.3% were afraid of getting sick and 6.6% were afraid of someone in the family getting sick with COVID-19. Concerns of a possible extended period of quarantine also showed variance between the beginning and the moment of the interview, with 3.3% and 33.6%, respectively. Other general concerns at the beginning and two months after the quarantine start are shown in Table 2.

**Table 1:** Orthodontic treatment concerns.

Orthodontic treatment concerns	n
Pain	3
Breakage	3
Oral hygiene	3
Appointment necessity	4
Length of treatment	5
None	12
Total	30

**Table 2:** General concerns.

General concerns	n (at the beginning of quarantine)	n (after 60 days of quarantine)
Lack of food	1	0
Lack of academic activities	4	5
Get sick	11	4
Family get sick	7	2
The quarantine period extends	1	11
Social distancing	1	0
Economic problems	0	2
None	5	6
Total	30	30

## DISCUSSION

Quarantine protocols have proven to be an effective solution for pandemic control.^11^ Social distancing has promoted a great impact in COVID-19 contagious rates, flattening the curve of infected people.^11^ Several countries have taken actions in an attempt to manage the economic effects.^3^ Nevertheless, the psychological impact of social distancing should also be considered, in view of the consequences that epidemic situations have in the quality of life.^2^ Considering the worldwide dissemination of COVID-19, studies focusing on physical and psychological responses are needed.

The relationship between pain and orthodontic movement is well known.^12^ ^,^ ^13^ There is a significant frequency of patients reporting pain some hours after the orthodontic appointment that can persist for a few days.^14^ ^,^ ^15^ Pain is usually related with new archwires or elastic force application.^14^ In this study, 16.67% of the patients reported dental pain produced by the orthodontic treatment, which is significantly less than previous studies^14^ ^,^ ^15^ (Fig 1). This is probably because the interviews were performed after at least 2 months after the last appointment. Pain was not related to tooth movement but to appliance breakage. Breakage urgency consultations are common during orthodontic treatment and assistance is usually immediately given.^16^ However, due to COVID-19, virtual assistance had to be offered.^7^ Despite pain and appliances breakage, almost the totality of the subjects showed no negative interference of the orthodontic treatment in daily life.

An emergency appointment is defined as any visit that was not planned but needed.^7^ In orthodontics, elastic modules loss, traumatizing archwires or attachments, archwire breakage, debonding of brackets and bands are the most common reasons for unscheduled appointment.^17^ However, orthodontic problems may be considered as urgencies and not as true emergencies and an unscheduled consultation might not be necessary.^7^ From the six participants that felt the need of an urgency appointment, only one relied on private consultation to improve oral hygiene. Seven patients (23.33%) reported that even feeling the need of an appointment they would only contact the professional by WhatsApp. This response was related to fear of a possible infection and/or economic reasons. 

Oral hygiene is severely affected by orthodontic appliances.^18^ Tooth brushing difficulties and increased favorable conditions for plaque accumulation may result in enamel demineralization and gingivitis.^19^ ^,^ ^20^ Considering that these situations are common in the orthodontic practice, a periodical follow-up is necessary.^21^ ^,^ ^22^ A 28-day interval between appointments shows a good biological response, and allows adequate control of the oral hygiene.^7^ Longer intervals between orthodontic visits require better patient compliance. Oral hygiene status was based on the self-perception of the patients and should be interpreted with caution, consisting in a limitation of this study. The results showed that even without orthodontic consultations, 36.67% of the patients considered that oral hygiene was improved during quarantine as a result of time availability for tooth brushing (Fig 2). Fifty-six percent reported that their oral hygiene did not change during the quarantine. Hygiene worsening was reported by few patients (6.67%) and was reported as a consequence of laziness. COVID-19 has paused orthodontic treatment follow-ups, nevertheless, most patients reported an adequate oral hygiene during quarantine. This could be related to the fact that patients were aware of the contagious risk during orthodontic appointments. Noninvasive remote patient monitoring has been reported in the healthcare field^23^ and might be usefully applied in the orthodontic field during the quarantine period. Reminder messages proved to be an effective motivational method, and orthodontists could use it during the quarentine time to improve patients oral hygiene.^24^ ^,^ ^25^ The use of self-photographs has also proven to be an effective tool in the control of oral hygiene.^26^ Photographs and videos should be used to improve patients’ compliance and mediate remote assistance during quarantine.

A limitation is the fact that the patients from the present study belong to the orthodontic clinic of a public university and might not be as demanding as private patients. However, the frequency of urgencies was possibly similar between public and private care. Regular orthodontic attention during COVID-19 quarantine was not possible, and a criteria for patients who need assistance is a fundamental preventive action.^7^ Digital apps may be useful to distinguish between urgencies and emergencies, so patients that really need attention can be assisted with minimum risk.^7^

## CONCLUSIONS

During COVID-19 quarantine, orthodontics patients demonstrated a low frequency of orthodontic appliance interference in daily life, and most of them reported no change in oral hygiene habits.

The frequency of appliance related pain or appliance breakage was 16.67% and 23.33%, respectively.

Patients’ main concern during quarantine was the fear of getting sick. They were also afraid of a longer extension of the quarantine period.
